# Factors preventing early case detection for women affected by leprosy: a review of the literature

**DOI:** 10.1080/16549716.2017.1360550

**Published:** 2017-08-30

**Authors:** Victoria Grace Price

**Affiliations:** ^a^ The Leprosy Mission England and Wales, Peterborough, Cambridgeshire, UK

**Keywords:** Gender inequality, health, stigma, treatment, leprosy, diagnosis, case detection

## Abstract

**Background**: Although leprosy can affect both sexes equally, it is globally reported that men are affected, or simply report, more often than females at the average ratio of 2:1. If cases are simply not being reported, women may be suffering in silence more often than men, and, therefore, understanding the social reasons for this in a number of countries could support the prevention of long-term disabilities caused as a result of leprosy.

**Objectives**: The objective of this review is to recognise the current academic literature surrounding the potential factors for late diagnosis of women affected by leprosy, giving possible explanations for the 2:1 gender disparity observed in case detection globally. It is hoped that health practitioners will become more equipped to recognise these barriers and ensure they are doing whatever possible to encourage women to report the early symptoms of leprosy.

**Methods**: The review used a systematic search process in order to identify gender-related publications using robust research, useful for gleaning a cross-cultural perception of issues women may confront on the prospect of a diagnosis of leprosy.

**Results**: Identifying 12 publications from just five countries, the review found there to be four overarching areas which may be considered barriers more often faced by women: societal stigma; women’s dependence and low status; self-stigmatising attitudes; and the gender insensitivity of leprosy services.

**Conclusion**: Stigma surrounding leprosy experienced from these four overarching areas can all be attributed to the later diagnosis of women affected by leprosy, in relation to their male counterparts. The need for future research surrounding the specific experience of women affected by leprosy is pressing.

## Background

The latest World Health Organization (WHO) [1] data on leprosy highlights a global trend between the numbers of new male to female cases. Table 1 displays these results among countries in Africa, the Americas and South-East Asia. Countries marked with an asterisk are those included within the data reviewed in this paper; the other countries are included as a comparison of the regions included within the review.

**Table 1.** WHO 2014 global leprosy update.

Region and country	No. of new cases detected (2014)	No. of females among new cases	% of females among new cases
**Africa**			
Nigeria*	2 983	1 277	43%
United Republic of Tanzania	1 947	701	36%
Democratic Republic of Congo	3 272	1 514	46%
**Americas**			
Brazil*	31 064	14 109	45%
Colombia	423	154	36%
Venezuela	513	174	34%
**South-East Asia**			
India*	125 785	46 379	37%
Indonesia*	17 025	6 370	37%
Nepal*	3 046	1 115	37%

Leprosy is a mildly infectious disease caused by the bacteria ‘Mycobacterium Leprae’ which, once contracted, inflames the skin and peripheral nerves, causing lasting and – without expensive reconstructive surgery – irreversible damage. The debilitating effects of the disease, caused by injuries to limbs affected by nerve damage, become long-term disabilities if the initial signs of leprosy are not detected early. It can affect all ages and both sexes as infection can happen at any time in life depending on opportunities and levels of contact [2]. The disease is believed to be transmitted via droplets, from the nose and mouth, during close and frequent contact with untreated cases. Cases often go untreated and undetected due to the slow multiplication of the bacteria, evidenced though the average incubation period of the disease being five years [3]. Once a major global public health concern, the disease is now considered eliminated, with less than one in every 10,000 people globally currently affected, however 200,000 people continue to be diagnosed annually [1] even though today the disease can be cured relatively easily through a course of Multi-Drug Therapy (MDT).

The data demonstrates that in every country listed above, and almost every country in the WHO global report, women are less represented in new cases detected. Other literature, such as Peters and Eshiet’s [4] study of over 2000 adults affected by leprosy in Nigeria, confirm that women have a much longer duration of illness between first symptoms and presentation for diagnosis, at almost twice as long. There is no medical evidence to suggest, within the author’s knowledge, that leprosy is more inclined to infect men over women. In some contexts, however, men may be more likely to come into contact with a greater social network outside of the household and, therefore, have an increased risk in contracting leprosy. Nevertheless, a higher risk of contracting leprosy has been demonstrated through frequent and intense contact groups [5], such as within the household. There are an increasingly large number of studies which attribute this disparity with social gender inequalities. The key findings of the majority of these studies will be discussed by the author in this review, with the expectation that gaining an understanding of why women are less inclined to present symptoms of leprosy might aid in the development of better interventions to more fully meet their needs and alleviate the barriers to treatment.

Studies pertaining from across the globe continue to show the disparity between the number of male and female patients being diagnosed with leprosy, as well as suggesting that women are more likely to present later for treatment. Nerve damage is a well-known outcome of delaying diagnosis and treatment of leprosy, with the potential to cause long-lasting physical disabilities [6]. Yet leprosy is as much a social issue as a medical one. The effects of leprosy can be felt not only in physical sensations, or lack thereof, but also socially, economically and emotionally. The stigma which surrounds leprosy and those who are affected by it in countries the world over can have such a detrimental impact on many aspects of life that many people feel admitting or acknowledging for themselves their condition to be too risky a decision. Leaving these early warning signs untreated can have long-term debilitating effects; therefore, ensuring early case detection through the removal of stigma is vital in order to see complete elimination of the disease worldwide.

Women can be triply disadvantaged with regard to health concerns, due to their gender, potential disabilities and the societal stigma which arises from them [7]. It is commonly understood by scholars within the gender and development field that gender equality can play a key role in the social and economic development of a country. Aikman and Unterhalter [8] draw on the work of Amartya Sen’s ‘capability approach’, stating that ‘gender equality entails *developing* the freedoms of all individuals, irrespective of gender, to choose actions, aspirations, and attributes that they have reason to value’ [9]. When women are not given equal opportunities to develop their freedoms in private or public life, one half of a community is silenced, unrepresented and unable to share the problems they face within their society to the whole community. When one gender is traditionally dominant over the other, dependence within this relationship can be created. If this dependant relationship breaks down, it can leave one party in a more trying position than the other, particularly when resources are scarce. Gender mainstreaming approaches have been developed by the likes of UNICEF, DFID (UK Department for International Development) and UN Women in an attempt to make the issues surrounding gender and development central to the work of policy makers and placing gender equality as a core value, at the heart of development practice and therefore society as a whole [10]. It is the aim of the author to make apparent the current research surrounding the experience of women affected by leprosy in typically patriarchal societies where they have historically and continue to hold lower social status; to explore the role this imbalance plays within efforts for early case detection of leprosy among women; and to point policy makers towards recommendations for a more effective gender approach in this field.

International strategies and networks such as ILEP (International Federation of Anti-Leprosy Associations) and WHO have acknowledged this disparity and are beginning to work towards increased early case detection of leprosy in girls and women. ILEP’s 2016–2018 Strategy [11] holds ‘increased early detection of leprosy, with a focus on women and children’ as its Strategic Goal number 1.1. WHO also have recently released their 2016–2020 Global Leprosy Strategy [12] with ‘stop[ping] discrimination and promot[ing] inclusion’ as its fourth core pillar, acknowledging the necessity to include gender barriers within the dialogue of social inclusion.

The objective of this review is to recognise the current academic literature surrounding the potential reasons for late diagnosis of women affected by leprosy, giving possible explanations for the 2:1 gender disparity observed in case detection globally. It is hoped that health practitioners will become more equipped to recognise these barriers and ensure they are doing whatever possible to encourage women to report the early symptoms of leprosy.

## Methods

### Study design

The decision to conduct a general literature review was made in order to assess the current state of research surrounding gender and leprosy, within a qualitative narrative. The amount of literature surrounding the topic rendered this type of literature review most useful to be able to give a fair overall impression of the themes highlighted within the literature and to point towards gaps and recommendations for future areas of research.

### Search strategy

A literature search of articles written in or translated into English was conducted to identify relevant articles in electronic databases. These databases included INFOLEP, MEDLINE, ScienceDirect, Scopus, JSTOR, PsychINFO and CINAHL Complete and Social Sciences Citation Index. The search was performed in May and June 2016. The same search terms were used in all the databases and these were various combinations of: leprosy; women; gender relations; stigma; female; experience; delay in treatment; health seeking behaviour; discrimination; low social status; economic dependency; early case detection; health workers; belief; tradition(al) – and the following countries (in which more than 1000 new cases of leprosy occur annually [13]) were also included individually alongside each search equation: Bangladesh; Brazil; DR Congo; Ethiopia; India; Indonesia; Madagascar; Mozambique; Myanmar; Nepal; Nigeria; the Philippines; Sri Lanka and Tanzania.

### Inclusion and exclusion criteria

A total of 12 articles were retrieved from the search which suitably met the criteria of being published primary or secondary research examining the experience of women affected by leprosy with a suitably robust methodology involving 10 or more participants within qualitative studies or over 100 participants within quantitative studies, and being no more than 20 years old. Any publications found which did not meet these criteria, directly focusing on the experience of women affected by leprosy or published before 1996, were excluded from the search in order to retain a 20-year relevancy to account for changing gender roles or attitudes within the countries of focus.

### Data extraction

A search protocol was used for this literature review which comprised a number of stages. Beginning with the question *‘What are the reasons for gender disparity in reporting figures among people affected by leprosy?’* in mind, the following stages were addressed:

1. Identifying potential studies via accessible and relevant databases using key words and search equations, and when necessary requesting from databases hard copies of articles not available online;
2. Using the inclusion and exclusion criteria to manually sift through potential studies and narrow down to only those meeting all requirements;
3. Assessing risk of bias of individual studies at the study level through rigorous manual assessment of methodologies, funding sources or any other known authorship information which may be relevant;
4. Examining remaining studies in depth to assess quality and manually extract relevant evidence to support the review question;
5. Developing a structure for analysis of data and identifying key themes; analysing these articles through the coding of elements that appeared repeatedly, and within the identified common themes;
6. Writing up findings within the decided structure for the review.

The data extraction protocol applied included identifying key themes within the 12 studies, extracting data which reinforced these themes and compiling a coded database in Excel. Focusing on the meaning of the data rather than the words, the review used manual open coding. These coded themes were utilised with the acknowledgement that there may be occurrences within the literature when the experiences shared by a respondent, for example, may overlap or fail to fall neatly into the defined themes presented by the author.

## Results

The literature search identified 12 publications [14–25] describing the experiences of women affected by leprosy, recognising potential female-specific reasons attributed for the late diagnosis of leprosy. Publications were only selected for the review if they dealt directly with women affected by leprosy and had a robust analysis of original primary data. The only countries subject to research included by the author in this review are India, Nepal, Indonesia, Nigeria, Ethiopia and Brazil. This sample of countries is due to the limited research surrounding the topic of the review, both generally and then within the even smaller sample available which meets the criteria of the review. This limitation has resulted in a selection of articles which address issues from countries with widely different cultures and traditions concerning the status and roles of women, and it is therefore difficult to generalise the experience of women affected by leprosy between them. However, despite the limited number of articles fitting the criteria of the review *some* general similarities can be drawn.

The findings indicate four areas which may aid in the explanation of the late diagnosis of women:

- Experience of Societal Stigma (12 sources)
- Low Status and Economic Dependence (12 sources)
- Inner Wellbeing: Self-Stigmatising Attitudes (8 sources)
- Gender Insensitivity of Leprosy Services (11 sources)

## Discussion

### Experience of societal stigma

Having been known to humanity for millennia, the long history of leprosy has enabled the incubation of traditional beliefs and stigmatising attitudes surrounding the disease to become embedded within societies around the world, due to the fear of incurability and the visible disability which results from nerve damage. In many parts of the globe a diagnosis of leprosy brings with it the fear of being ostracised by one’s community, often due to a severe lack of medical awareness, particularly in remote areas where medical resources are scarce. While it is true that this societal stigma can affect both men and women, there are particular aspects of the cultural expectations of women in the countries reviewed, which result in disproportionate prejudice and pressure toward women when they are additionally affected by leprosy.

#### Traditional or religious societal beliefs about leprosy

Strong traditions, such as those summarised in articles [14,18,19] from Indonesia, Nepal and Brazil, present leprosy to have supernatural origins, such as a curse from God or ancestor for punishment of sins, or as the result of witchcraft. Where these historic traditions surround leprosy, the community stigma attached to those affected can be huge, yet these attitudes are not necessarily gender neutral. The above studies and others [17,20], including one in India, suggest that there is an expectation for women to visit a traditional healer before, if not solely, to cure the spiritual or traditional problem of leprosy. The extent of this finding varies throughout the articles. However there is a predominant impression, made explicit through one study in Indonesia [17], that women are more inclined to use religion or spirituality as a method of coping with societal stigma and finding solace throughout the discrimination, although the literature is not conclusive how much more this is for women than men. Schuller et al.’s [19] study in Indonesia makes evident that women are never invited to attend community gatherings due to their fear of the affected person, yet the community had no problem with allowing access to religious meetings. Here, the message about leprosy was clear from the religious leader: it is not a curse, but a test from Allah, which can and should not be rejected by man.

Other reasons attributed to visiting traditional healers before a medical clinic, as described by the literature, include: the lack of confidence in Western medicine and wariness of its effectiveness [16], but also, as found by two studies in Brazil [14] and India [21], the lack of knowledge surrounding the side-effects of the treatment used to treat leprosy, Multi-Drug Therapy (MDT). De Oliveria [14] in particular comments on the fear experienced by women in Brazil concerning the affect the treatment has on the appearance of the skin. The darkening of the skin is not only undesirable for women in terms of beauty standards, but also unwanted due to the social burden of required explanation.

#### Medical misconceptions about leprosy

Lack of information and communication surrounding leprosy, other false beliefs about how the disease can be contracted, and its incurability are common findings within the literature, particularly within all four of Varkevisser et al.’s case studies [18,19]. Misinformation is found to often be the root cause of perceivably justified societal stigma, for example, that leprosy is hereditary, was a belief found in Indonesia. This misconception can drastically reduce women’s marital chances, being dismissed by prospective spouses for fear of producing leprosy-affected children, a finding not attributed to the experience of men [18,19]. Fear of infection is another norm found to fuel societal stigma. Believing that simply sharing a meal or passing someone on the road who has ever been affected by leprosy, are examples of the extent to which leprosy is feared in many of the observed communities. All 12 articles comment, to varying degrees, on collective stigma’s outcome of isolation and exclusion of patients from the community, demonstrating the cross-cultural acceptance of this behaviour.

#### Stigmatisation from families

The permissibility of this exclusion was found to be doubly demanding for women due to their role as mothers and providers, throughout the literature. Being excluded from the home, unable to even touch their own children and fearing what their children must think of them, are reported to be some of the most difficult aspects of external stigmatisation experienced by women, particularly in India [20,21], Indonesia [19] and Brazil [14]. In research studies in Nepal, Van’t Noordende et al. [22] and Try [23], describe the fear that women have of being in their community without ever having been married, or having experienced divorce, stating that being married is an important pillar of Nepali society. Van’t Noordende et al. [22] suggest that women were far more likely than men to be deserted by their spouses on diagnosis, while Try [23] goes on to explain the undesirability of divorce and its ability to degrade a woman’s status in society. In one case study she demonstrates the choice women can be left with, between enduring the physical and verbal abuse of a husband and having to ‘return as a disgrace to my parents’ house’.

### Dependence and low status of women

The review identified that all but one of the included studies demonstrate how the status of women in India, Nepal, Ethiopia, Brazil and Indonesia can unequally burden women if they are to discover leprosy symptoms. The social burden of simply being a woman in these situations can result in unequal access to services, even if they are immediately available. Rao et al.’s [20] study in India found a clear gender disparate delay in initiating and continuing to administer formal treatment, observing that the low social status of women in India directly results in greater suffering for women. They reference extreme dependence and discrimination in every area of their lives as a defining characteristic of the poor situations in which women can find themselves.

#### Physical dependence on others

In India and Nepal, three studies present the difficulty women with low socio-economic status face if they wish to independently travel to treatment clinics or leprosy centres. John et al. [15] suggest, from their data in India, that women had to wait until their husbands or guardians felt it was necessary to go to hospital. The Nepali case study within Varkevisser et al.’s study [18] details how women’s dependence on their husbands or in-laws for any decision affects their ability to have their wishes heard and acted upon. Acknowledging that men and women both attribute a mother-in-law as a daughter-in-law’s worst enemy, they comment that some men confessed a women’s virilocality is the principle reason for stigma to be applied to female patients more severely than males. Women in these situations are judged solely on their ability to produce healthy children and conduct domestic duties well, and if they are unable, it is understood that they are no longer necessary for the household. This opinion is very much brought to light within the detailed case studies in Try’s research in Nepal. One man goes as far as to ask rhetorically: ‘If men refused to help her, then how could she survive? Men can survive with a small piece of cloth but female can’t. Women suffer more … husband is everything for a wife’ [23]. This dominance over critical decisions and household members, according to the research findings of this study, allows men to retain more respect than women if they face the stigmatisation of leprosy.

#### Economic dependence on others

In Varkevisser et al.’s [18] analysis of the four studies from Nepal, Nigeria, Indonesia and Brazil, it was found in that in all four areas of research, men were far better off financially. Ramos et al.’s [25] study in Ethiopia found that in their retrospective study of patient records, far fewer women had been admitted to the clinic with neuropathic skin ulcers, even though it is one of the most common results of leprosy. The authors suggest that the economic burden that comes with accessing the clinic and continuing the care for wounds could likely be ascribed to this finding. Kumar et al. [16] also attribute the actual or perceived cost of travel to their finding that, in their study area in Nepal, men were twice as likely to complete treatment as women. In Try’s [23] analysis of Maithili culture in Nepal, she notes how expected gender roles for men as wage earners and women as carers and home keepers are strictly upheld; and in accordance with these expectations, women affected by leprosy experience more restrictions on their daily activities due to this strict binary. John et al. [15] propose that women affected by leprosy in India suffer more adversely than men directly due to this lack of autonomy and the financial constraints upon them.

#### Impoverishment through divorce

In many countries such as India, Nigeria and Indonesia, it is still legal or traditional for one spouse to expect and be granted a divorce on the discovery of leprosy in the other, and as an Indonesian case study [18] notes, particularly when deformities begin to occur. Some of the reviewed articles consistently draw on this theme, detailing the unequal experience women face if they are diagnosed with leprosy before marriage, or in the event of a divorce if diagnosis occurs afterwards. Disabilities and stigma surrounding the disease disproportionately affect women. Studies reviewed with a focus on marital relationships in India [24] and Brazil [14] found that women were more likely to be single, divorced or living without their partner. Due to the previous findings of women’s dependence on men, both physically and economically, the literature goes on to illustrate how being dehabilitated from the family group or divorced places women in a very unstable social and economic position. In Vlassoff et al.’s study in India, women reported having experienced pressure to leave the household more frequently than men. In societies where women’s domestic work will constitute the majority of their economic contribution, the inability to complete tasks due to disability or stigma can render women worthless to their families. As one woman exclaimed [21]: ‘Why would they take us if our hands were wasted? They have married our hands which cook and clean and sweep!’

#### Physical and sexual abuse

Due to the low status and disempowerment of the women within the communities included by the author in this review, if they are unfortunate enough to live with a family who cannot accept the disease and do continue to stigmatise them, they are often forced to choose to either stay in the household where there may be emotional or physical abuse or neglect; or, to leave and potentially be left with little to no livelihood opportunities by themselves. For the many women included within the reviewed research, this was not a particularly difficult decision to make. In Varkevisser et al.’s Nepali case study [18], even despite deformities, it was found that many spouses would remain loyal, although women more often than men. Three studies [14,18,22] highlight the sexual experience of couples once the wife contracts leprosy. All three papers’ findings agree that women experience less sexual freedom than men, particularly if they have leprosy. For example, in Nigeria, men have more freedom to abstain from sexual intercourse on the leprosy diagnosis of their wives, than women would if it were their husbands who were diagnosed [18]. In Nepal and Brazil alike, more women affected by leprosy said they did not find sex an important part of their marital relationships, but their views were not considered important or taken into account by husbands. Women simply had to be ready when their husbands were ready. If they blankly refused to engage in sexual intercourse, their husbands enjoyed more freedom to engage in sexual activity elsewhere [14,22].

#### Limited mobility outside of the home

The expected gender roles of women to be good mothers and wives, presiding over child care and domestic duties, was found in two studies to be the main cause of women’s limited mobility outside the household [15,23]. John et al. [15] describe that in India, women’s access to medical facilities often depended on whether or not they had the time to visit, having to complete their household chores before setting out and after their return. It was highlighted that frequently service points kept women waiting, conflicting with their domestic work and lowering their social worth. Varkevisser et al. [18] comment that in all four study areas (Brazil, Indonesia, India and Nigeria) men were much more mobile than women, and therefore more able to travel further distances without the pressure to return as soon as possible. Other prominent research [26–28] outside of this review also points to the conclusion that women from low socio-economic backgrounds often have a much smaller pool of social contacts due to their limited mobility, resulting in far narrower social capital and inability to utilise the support or other resources a wider scope of contacts may be able to afford. Another finding which may also be linked to this social dependence is the educational status of women included within the research. Schuller et al. [19] suggest that disabilities and stigma surrounding women affected by leprosy in Indonesia directly resulted in lower educational levels. In Nepal and Brazil level of education was found to differ greatly between men and women. Low literacy rates among women or none at all, combined with the heavy workload and young marriage age were attributed by Varkevisser et al.’s Nepali case study [18] to the poor knowledge and awareness of clinical signs of leprosy and its treatment. An alternative discussion from de Oliveira’s [14] study found that in Brazil, men affected by leprosy were able to leave employment if their disabilities restricted their work, whereas women were less able due to the necessity of the domestic economy. This gendered distinction would result in the difficulty for women to engage in any activities outside of the household, such as non-formal educational projects or programmes.

### Inner wellbeing: self-stigmatising attitudes

The review identified eight sources which establish themes surrounding the self-stigmatisation and mental wellbeing of women affected by leprosy. These themes range from hints towards negative self-attitudes as a potential reason for late diagnosis, to conclusive findings indicating strong correlations between internalised stigma and unwillingness to disclose their presumed health status. Kumar et al.’s [16] article outlines the gender differences in factors associated with treatment completion in Nepal, and notes that completion behaviours may relate to factors such as accepting the condition as final and unchangeable, with no urgent need for treatment. This concept could relate to the earlier point surrounding the belief that the disease is a curse from a God, evil spirit or ancestor, however it may also be attributed to the notion that women in cultures such as Nepal don’t view themselves as individuals, but as an integral part, if not the unassuming cornerstone, of the collective that is the family or household. This lack of attention to their own inner wellbeing can increase stigmatising attitudes of women towards themselves, lowering self-esteem and intrinsic worth as a human being.

#### Women as more susceptible to mental illness as a result of diagnosis

Peters et al. [17] pick this concept up in more detail, considering narratives around concealment of leprosy in women in Indonesia. They studied 53 women and found that the women who had concealed their illnesses the most more frequently reported feeling sadness, shame, low self-esteem and depression, and that these feelings often result in self-isolation. Following this, the authors observe that it is not enacted stigma of others which leads women to conceal their disease, but anticipated stigma, which may or may not be received in reality. Women who continued to conceal their disease due to this fear instead increased negative feelings, emotions and internalised stigma. De Oliviera [14], Schuller et al. [19] and Rao et al. [24] all maintain the notion that women experience a much higher degree of self-stigmatising attitudes. These studies pertain from Brazil, India and Indonesia respectively; however, all equally point towards the acknowledgement of lowered levels of self-esteem, guilt and anxiety for women as an outcome of a leprosy diagnosis. In Schuller et al.’s [19] study exploring the experiences of women with disability in Indonesia, women with physical disabilities due to the late diagnosis of leprosy had a much higher number of psychological problems compared to women with other disabilities. The women themselves remarked that they were too embarrassed to let other people see their disabilities, and that they preferred to stay inside away from others altogether when possible.

#### Anxiety over identity loss

Another issue highlighted by the literature is that of the loss of identity for women when they become unable to perform household duties. That disabilities relating to leprosy are able to impact gender-prescribed roles in the household is yet another cross-cultural observation. In Brazil, de Oliviera [14] reports that women experience guilt when physical limitations are placed upon them to be able to contribute to household activities such as cooking or looking after children. She notes that a woman’s inability to fulfil family roles can result in emotional breakdown and a sense of being stripped of the attributes which they themselves deem as necessary in order to be a competent woman. Three studies from India and Nepal [19–21] confirm this finding, attributing a limitation on family roles towards the decrease in self-esteem and self-worth as mentioned by other authors. One [19] suggests that women affected by leprosy feel guilt and shame when they are unable to contribute to the household, and another [21] goes even further to attribute this redundancy to a cause for women to want to, on their own accord, leave the household altogether, causing potentially serious economic problems.

### Gender insensitivity of services

The final theme addressed as a barrier to early case detection by the literature is the experience women may have had in relation to leprosy services. Eleven sources make reference to the inadvertent difficulties and constraints women may face when attempting to access professional help, due to previously mentioned tasks and roles expected of women, which may not have been taken into account by service providers. One aspect of this difficulty can be the physical infrastructure of leprosy clinics or services. Whilst not a female-specific issue, two articles noted that the lack of roads suitable for motor vehicles in order to provide access to leprosy services in remote regions of Nepal. When services are difficult to access, particularly in dry and rainy seasons, and when resources within services are basic at best, women may find it even more demanding to convince others to accompany them in their urgent time of need [16,18]. Varkevisser et al. [18] also note that in Nepal, privacy for women within clinics was a problem, where it was reported that health facilities were so small that several activities had to take place in one room, unsurprisingly making patients reluctant to undress.

Two studies from India [15,24] echo the urgent need to ensure privacy during physical examinations, or female-only bathrooms, as a way to ensure an acceptable standard of provision for women.

#### Staff–patient relationships

Staff–patient relationships are another aspect of leprosy services which must be considered through a gender lens, as relationships with the opposite sex are found in three articles [15,16,18] to be a potential problem in the diagnosis of women affected by leprosy. In an overview of three studies in Indonesia, Nigeria and Nepal, Varkevisser [18] notes how male staff formed the majority in all three cases, yet even in pockets where women were overrepresented within staff, both sexes encountered difficulties approaching and investigating patients of the opposite sex. John et al. [6] mention that of their study in India, given that 60% of women had delayed seeking medical care and concealed their disease, the need is urgent for services to be sensitive to the requirements of women through the employment of enough trained female staff. Interestingly, they also note, and are seconded by Kumar [16], that the quality of the relationship between staff and patient is equally important in order to uphold the retention of women affected by leprosy within their treatment cycle. They recommend that ensuring enough time is given to each patient to listen to their individual problems and counsel them would ensure that patients are not disappointed or feel let down by the service.

#### Patient and family counselling

The inadequacy of patient and family counselling is mentioned within five of the reviewed articles [15,18,21,22,25], with all four case studies from Varkevisser et al.’s study [18] reporting this inadequacy in services. Patients would receive some information about treatment and curability of leprosy, but little to nothing about the symptoms, causes or infectiousness. This is particularly pertinent for women due to their intrinsic position within the family. If the family (the spouse and possibly in-laws) were to receive professional and comprehensive counselling about the implications of a leprosy diagnosis, women may be more empowered to continue to live freely without fear of discrimination from her own family, preventing many of the reported problems [15,22]. The understanding that services do not provide family counselling after a diagnosis of leprosy could be a huge factor contributing to the decision process of a woman with leprosy debating engaging in treatment or not. As Vlassoff et al. [21] report from India, the indifference faced by women affected by leprosy from their families caused them great suffering. Providing information to families is of utmost importance in order to help them understand the crucial role they play in aiding patients to cope and recover, as well as minimising the risk of patients being dehabilitated.

#### Information, education and communication

Six articles present information, education and communication to have a huge role to play in the elimination of community-based stigma which surrounds leprosy [15,16,18–20,22]. In the same vein as family counselling, educating the entire community about the realities of the disease is believed to be a vital role that leprosy services must adopt. The curability of the disease must be explained in more detail, and in the terms of the community in a culturally acceptable way [15,22], and with the participation of patients and ex-patients themselves [18]. Myths about the speed of improvement and side effects of taking MDT is an issue the literature repeatedly recommends need to be addressed. The lack of awareness about leprosy and being able to identify it, in one study, was a leading reason for the delay in initiating formal treatment among women in the research area in India [20].

#### Improved education of health professionals

In some cases, the literature shows that it may not be the lack of potentially beneficial health education within the community, but the lack of adequate training about leprosy on the part of health professionals, which leads to a lack of leprosy diagnosis [14,16,18,21]. Whether that be private or government doctors who have not received training on leprosy [18], or counsellors with little knowledge about the side effects of MDT [14,21], it is clearly beneficial to any person affected by leprosy (but particularly women who can face many other hurdles in diagnosis and treatment) that health professionals are robustly equipped to manage a leprosy diagnosis effectively from start to finish.

The inability of services to take into account the special needs of women, expecting them to have the same expectations and experience of services as men despite the very different way society treats them, clearly has an impact on the acceptability of these services. Gender bias is shown throughout the reviewed literature to have an important implication for detection, treatment and control of the disease, and should therefore be taken seriously when planning and monitoring health/education interventions.

## Limitations

It must be acknowledged that in choosing to conduct a general literature review there is no scope for a deep analysis of the data collected, but simply a presentation of recurring themes found within the literature. Whilst the author acknowledges this as a strong limitation, what is presented is hoped to be as fair and unbiased a representation of the literature as possible with a singular author. For future research, there is perhaps enough literature to conduct a more rigorous and scientific approach such as a scoping review. Due to time and professional resource constraints, however, a general literature review was conducted in this instance, with the strength of this review method lying in its ability to build on previous work and allowing for consolidation [29].

In order to identify loss of information, it is acknowledged that the review is limited only to academic information found through the databases listed below. No attempt has been made to include any literature in languages other than English or translated into English, or any grey material such as documents or information from non-governmental organisations (NGOs) or other organisations. It is worth noting that other documents of this nature would be worth investigating within any future, more extensive, scientific research.

## Conclusion

As summarised by the authors throughout this review, there is clearly plentiful evidence for the very distinct way women experience a diagnosis of leprosy. Stigma surrounding leprosy experienced from the wider community, resulting in increased self-stigma, low status and economic dependence and the potential gender insensitivity of services can all be attributed to the later diagnosis of women affected by leprosy, in relation to their male counterparts. Whilst these four areas are more than likely not the only areas in which women face increased difficulties on a diagnosis of leprosy, they may be a good starting point in which organisations working in this area may be able to tailor interventions. In a literature review of interventions available to promote early case detection of leprosy, Nicholls et al. [6] note that it is outstanding that there are no specific interventions working to address these distinct issues, which demonstrates a gap between research and practice. That said, the need for future research surrounding the specific experience of women affected by leprosy is pressing. In order to supply practitioners with a robust evidence base of the barriers faced by women in their own specific communities, research is needed to best tailor future interventions and ensure best practice. Much can be done to further the case for women affected by leprosy without increased expenditure, but by simply ensuring that the lessons are learnt concerning how local culture can influence perceptions of women and to address their needs more effectively [15,23].

## Paper context

Although leprosy can affect both sexes, it is reported that men are affected, or report, more often than females at the average ratio of 2:1, with no scientific evidence in existence for this disparity. This paper reviews literature from six countries and finds four overarching themes which may point towards socio-cultural, gender inequality-based explanations; suggesting practitioners consider findings when formulating projects for increased gender-awareness and earlier case detection among women.
